# Same-Session Hybrid Embolization and Microsurgery Versus Endovascular-Only Treatment for Cerebral Arteriovenous Malformations: A Retrospective Single-Center Comparative Cohort Study

**DOI:** 10.3390/jcm15176545

**Published:** 2026-08-25

**Authors:** Hasan Bilen Onan, Bişar Akbaş, Ferhat Can Pişkin, Kadir Oktay, Kerem Mazhar Özsoy, Duygu Özgül Özesen, Erol Akgül

**Affiliations:** 1 Neuroradiology Private Practice, 01060 Adana, Türkiye; 2 Department of Radiology, Faculty of Medicine, Cukurova University, 01330 Adana, Türkiye; bisarakbas20@gmail.com (B.A.); ferhatcpiskin@gmail.com (F.C.P.); duyguozgul.d@gmail.com (D.Ö.Ö.); 3 Department of Neurosurgery, Faculty of Medicine, Cukurova University, 01330 Adana, Türkiye; koktay@cu.edu.tr (K.O.); mazhartac95@hotmail.com (K.M.Ö.); 4 Department of Radiology, Faculty of Medicine, Istanbul Medipol University, 34214 Istanbul, Türkiye; eakgul@medipol.edu.tr

**Keywords:** arteriovenous malformation, embolization, microsurgery

## Abstract

**Background/Objectives:** Same-session hybrid treatment joins embolization to immediate microsurgical resection under one anesthetic, closing the interval of partial devascularization that follows staged embolization. Comparative data are scarce. We set out to compare angiographic, functional and safety outcomes after hybrid and after endovascular-only treatment and to judge how far any difference reflects case selection and calendar period rather than the strategies themselves. **Methods:** We reviewed 43 consecutive patients treated for cerebral arteriovenous malformation at one center between May 2012 and December 2024. Nineteen received same-session hybrid treatment and 24 embolization without same-session surgery. The primary endpoint was complete obliteration on six-month angiography. Differences are reported with 95% confidence intervals, and odds ratios were estimated using Firth penalized regression, since both principal comparisons showed complete separation. **Results:** Hybrid patients were younger (median 31.0 versus 42.5 years; *p* = 0.040), with similar sex distribution and Spetzler–Martin grade. Complete obliteration was reached in all 19 hybrid patients (100%) and in 14 of 24 endovascular-only patients (58.3%), representing a difference of 41.7 percentage points (95% CI 17.6–61.2). All four deaths occurred in the hybrid group (21.1% versus 0%; *p* = 0.031). Complications (15.8% versus 12.5%) and favorable outcome (mRS 0–2, 68.4% versus 75.0%) did not differ. The strategies also belonged to different periods. Eight of 19 hybrid but 21 of 24 endovascular patients were treated before 2019 (*p* = 0.003), as were three of the four deaths. **Conclusions:** The hybrid treatment obliterated every malformation but accounted for every death, with no difference in functional outcome. Because the groups served different clinical purposes in different eras, these observations are hypothesis-generating rather than grounds for changing practice.

## 1. Introduction

Brain arteriovenous malformations (AVMs) are congenital tangles of dysplastic vessels in which arterial blood passes directly into draining veins through a nidus, without an intervening capillary bed. They are uncommon: detection rates run at roughly 1.1 to 1.3 per 100,000 person-years and adult prevalence at 10 to 18 per 100,000, with men and women affected at broadly similar rates and diagnosis usually made between the third and fifth decades [1]. Because the population is young, both the risk of an untreated lesion and the cost of any treatment complication are carried for decades.

Hemorrhage is the dominant concern. Pooled across 4240 patients, the annual rate is about 2.3%, roughly 1.3% for lesions that have never bled and 4.8% after a first bleed, with prior rupture, deep venous drainage and associated aneurysms the most consistent predictors [2]. Ten to fifteen percent of these hemorrhages are fatal, and a further 30% to 50% of survivors keep a permanent deficit [1]. About half of patients therefore present with hemorrhage. Seizures come next, particularly with cortical lesions, and the rest present with headache, a progressive deficit attributed to steal or venous hypertension, or are found incidentally.

Computed tomography is used acutely and magnetic resonance imaging otherwise, with the latter showing the nidus as a cluster of flow voids and its relation to eloquent cortex. Digital subtraction angiography (DSA) remains the reference standard, since only dynamic imaging resolves the feeding pedicles, the internal architecture of the nidus, flow-related aneurysms and the timing of venous drainage. Those features determine what can be done. Microsurgical resection gives immediate and durable cure and is preferred for accessible Spetzler–Martin grade I and II lesions, where obliteration exceeds 95% [3]. Radiosurgery suits small deep lesions but works over two to three years, during which bleeding risk continues [4]. Embolization alone reaches only 45% to 61% obliteration when used with curative intent [5,6]. Conservative management remains reasonable for unruptured high-grade lesions, a view strengthened after ARUBA found worse outcomes with intervention. However, that trial drew criticism for short follow-up and for how heavily it relied on embolization [7,8].

Embolization is frequently staged, which limits the embolic volume delivered at one sitting and lets the circulation adapt to redistributed flow. The price is an interval of weeks or months during which a partly occluded nidus sits exposed to altered pressure, and delayed rupture in that window is well described [5,9]. Same-session hybrid treatment abolishes the interval: embolization and resection are performed consecutively under a single general anesthetic, with the patient being transferred directly from the angiography suite to the operating room without waking and intraoperative angiography confirming the result before the patient leaves the table [10,11,12]. In exchange, the entire procedural risk is compressed into one admission.

Published hybrid series are small and single-center, and usually report the hybrid cohort alone or against historical surgical controls. Missing is an account of how hybrid treatment performed alongside the endovascular pathway it displaced inside one institution. We aimed to describe angiographic, functional and safety outcomes after each approach over twelve years and to measure how far the differences are entangled with case selection and calendar period. The groups were not competing answers to one clinical question: the hybrid group holds patients chosen for definitive surgical cure, while the endovascular-only group mixes curative intent with embolization planned as one step of a longer course. Comparison of obliteration between them is asymmetric by construction, and we framed the analysis and conclusions accordingly.

## 2. Materials and Methods

### 2.1. Design and Ethics

This was a retrospective, single-center, non-randomized comparative cohort study of patients treated for cerebral AVM between May 2012 and December 2024. The institutional review board approved the study (decision no. 37, February 2026) and waived individual consent given the retrospective design. Reporting follows STROBE.

### 2.2. Eligibility

Inclusion criteria: Cerebral AVM confirmed on DSA, magnetic resonance angiography or computed tomographic angiography; treatment at our institution by either same-session hybrid embolization with microsurgical resection or embolization without same-session surgery; pre-treatment imaging sufficient to assign a Spetzler–Martin grade; a documented treatment record; and at least one post-treatment assessment with a recorded modified Rankin Scale (mRS) score.

Exclusion criteria: Baseline imaging too incomplete to characterize angioarchitecture; no post-treatment angiography and no follow-up mRS; primary microsurgery without any endovascular component, since such patients belong to neither group; radiosurgery as the sole first modality; a diagnosis other than a true nidal AVM; and treatment elsewhere with only local follow-up.

**Figure 1:**
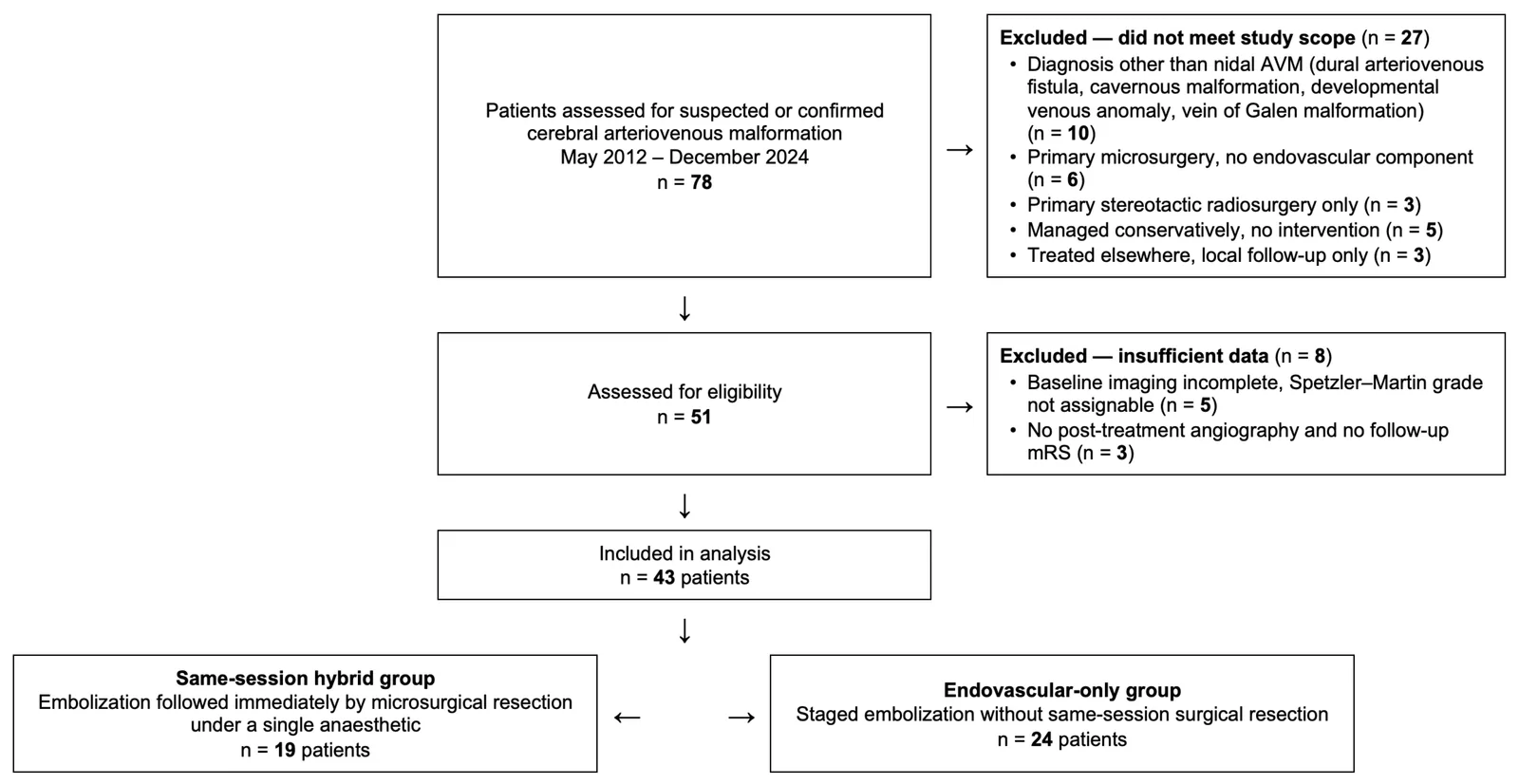
Flow diagram of patient screening, eligibility assessment and allocation to the two treatment pathways. Arrows indicate the direction of patient flow through screening, exclusion, eligibility assessment, inclusion, and treatment-group allocation.

Every patient meeting these criteria was included, so the cohort is consecutive within each pathway (Figure 1).

### 2.3. Baseline Status and Comorbidity

The unit of analysis is the patient. A total of 43 consecutive patients were included: 19 treated with same-session hybrid embolization and microsurgical resection and 24 treated endovascularly.

Baseline neurological status was the presenting mRS, dichotomized at >2; this threshold was exceeded in seven of 43 patients (16.3%). Medical comorbidity was documented in the notes but was never coded consistently over twelve years, because the earlier part of the cohort predates our electronic coding and reconstructing a Charlson index from free text would have produced a variable of questionable reliability. This matters because comorbidity did influence allocation: patients deemed to carry high anesthetic or surgical risk were steered toward embolization.

### 2.4. Allocation and Calendar Period

Allocation was neither randomized nor contemporaneous, following the multidisciplinary team’s judgment together with the institution’s changing technical capability. Hybrid treatment was favored for surgically accessible supratentorial lesions in patients with acceptable operative risk and a clear indication for single-session cure. Endovascular-only management was chosen more often for deep or infratentorial lesions, elevated surgical risk from age or comorbidity, embolization preceding planned radiosurgery, and patients declining open surgery.

Hybrid procedures ran throughout the study period but their frequency shifted after a standardized institutional protocol for same-session treatment was introduced in 2019, while endovascular-only treatment moved the other way. This created a marked temporal imbalance between treatment groups.

### 2.5. Technique

Endovascular work was performed on a flat-panel biplane system (Artis zee biplane, Siemens Healthineers, Forchheim, Germany) by experienced neurointerventionalists. Two experienced cerebrovascular neurosurgeons performed microsurgical resections according to standard microsurgical principles. Ethylene-vinyl alcohol copolymer alone was used in 16 of 19 hybrid patients and 18 of 24 endovascular-only patients, *n-butyl cyanoacrylate* alone in 2, and both together in 3 hybrid and 4 endovascular-only cases. Devascularization, estimated fluoroscopically, exceeded 75% in 14 of the 24 endovascular-only patients.

In hybrid cases embolization was performed under general anesthesia immediately before craniotomy, aiming to occlude deep and surgically inaccessible feeders and to reach at least 70% nidal occlusion where safe. The patient was then transferred, still intubated and under the same anesthetic, to the operating room for craniotomy and resection. Endovascular-only embolization was staged with the interval judged case by case. Twelve of 24 such patients needed more than one session, compared with 3 of 19 hybrid patients. Resections followed standard principles, with the principal draining vein preserved until the arterial supply was interrupted and intraoperative angiography performed before closure.

### 2.6. Outcomes

Complete angiographic obliteration, the primary endpoint, meant no residual nidus, shunt or early venous filling on DSA at six months. It does not mean the same thing in both groups. For hybrid patients it reflects a strategy meant to be definitive in one session. For endovascular-only patients it reflects status six months after the endovascular course, which for some was the end of treatment and for others an interim assessment before planned radiosurgery. The comparison therefore asks whether the assigned strategy had achieved cure by six months, not whether the two modalities cure equally well given further treatment. Interpreted in this way, the comparison inherently favors the hybrid strategy.

Treatment-related complications were any new neurological deficit, any imaging-confirmed cerebrovascular event attributable to treatment, or a rise of at least one mRS point at discharge. All-cause mortality covered death during the index admission and at any later point. Functional outcome was the mRS at last follow-up, split into favorable (0–2) and unfavorable (>2), with death counted as unfavorable. Each adverse event was counted once, with events resulting in death reported separately in the corresponding mortality rows.

### 2.7. Statistical Analysis

No a priori sample-size calculation was performed; the cohort is every eligible patient in the period, so its size was set by disease frequency and caseload. Precision is therefore reported throughout as confidence intervals rather than as post hoc power. With four deaths the events-per-variable ratio is 4, well under the conventional minimum of 10, and both principal comparisons show complete separation, so all adjusted models used Firth’s penalized likelihood and are exploratory.

Continuous variables are median and interquartile range, compared by a two-sided Mann–Whitney U test with rank-biserial correlation as the effect size. Categorical variables are counts and percentages compared by Fisher’s exact test, used throughout because expected cell counts were mostly below five. Proportions carry Wilson score intervals, differences carry Newcombe hybrid score intervals, and odds ratios came from Firth regression with profile penalized-likelihood intervals. Survival was estimated by Kaplan–Meier and compared by log-rank test, with peri-procedural deaths assigned one day. Confounding was addressed using Firth-penalized logistic regression, adjusting for treatment group and calendar period and, separately, for age and Spetzler–Martin grade. Propensity-score methods were considered but not pursued because the positivity assumption was violated, preventing reliable covariate balance between treatment groups. Tests were two-sided at *p* < 0.05 without correction for multiplicity in Python 3.11.

## 3. Results

### 3.1. Cohort and Distribution over Time

A total of 43 patients met the criteria: 19 hybrid patients and 24 endovascular-only patients (Figure 1). Median follow-up was 41.5 months (IQR 14.5–65.2) against 53.0 months (IQR 16.5–83.2; *p* = 0.413), with duration missing for five patients.

The treatment strategies were applied predominantly during different calendar periods. Eight of 19 hybrid patients (42.1%) but 21 of 24 endovascular-only patients (87.5%) were treated between 2012 and 2018 (*p* = 0.003), and the median year of treatment was 2019 against 2014. Calendar period was by far the most imbalanced variable in the dataset, so comparing the groups also compares two eras of practice, and the two cannot be cleanly separated.

### 3.2. Baseline and Angioarchitectural Characteristics

Table 1 gives baseline characteristics. Hybrid patients were younger, at a median 31.0 years (IQR 18.5–37.0) compared with 42.5 (IQR 32.8–52.0), the only baseline difference reaching significance (*p* = 0.040, r = −0.37). Sex, baseline mRS > 2 and presenting symptoms were comparable.

Angioarchitecture was closely matched. Median Spetzler–Martin grade was 3.0 against 2.5 (*p* = 0.750), and grade III-IV lesions were almost equally frequent at 10 of 19 (52.6%) and 12 of 24 (50.0%; *p* = 1.000). All 19 hybrid patients had supratentorial lesions, compared with 19 of 24 endovascular-only patients (*p* = 0.056); the five infratentorial lesions in the series were all managed endovascularly, and other angioarchitectural comparisons were non-significant. Hybrid patients needed fewer embolization sessions and stayed a median of 10.0 against 3.0 days in hospital (*p* = 0.004).

### 3.3. Complete Angiographic Obliteration

All 19 hybrid patients achieved complete obliteration at six months (100%) against 14 of 24 endovascular-only patients (58.3%); a risk difference of 41.7 percentage points (95% CI 17.6–61.2); a Firth odds ratio of 28.2; and *p* = 0.001. The very wide upper confidence limit reported in Table 2 follows from complete separation: with no failures in the hybrid group, the data cannot bound the effect from above.

**Figure 2:**
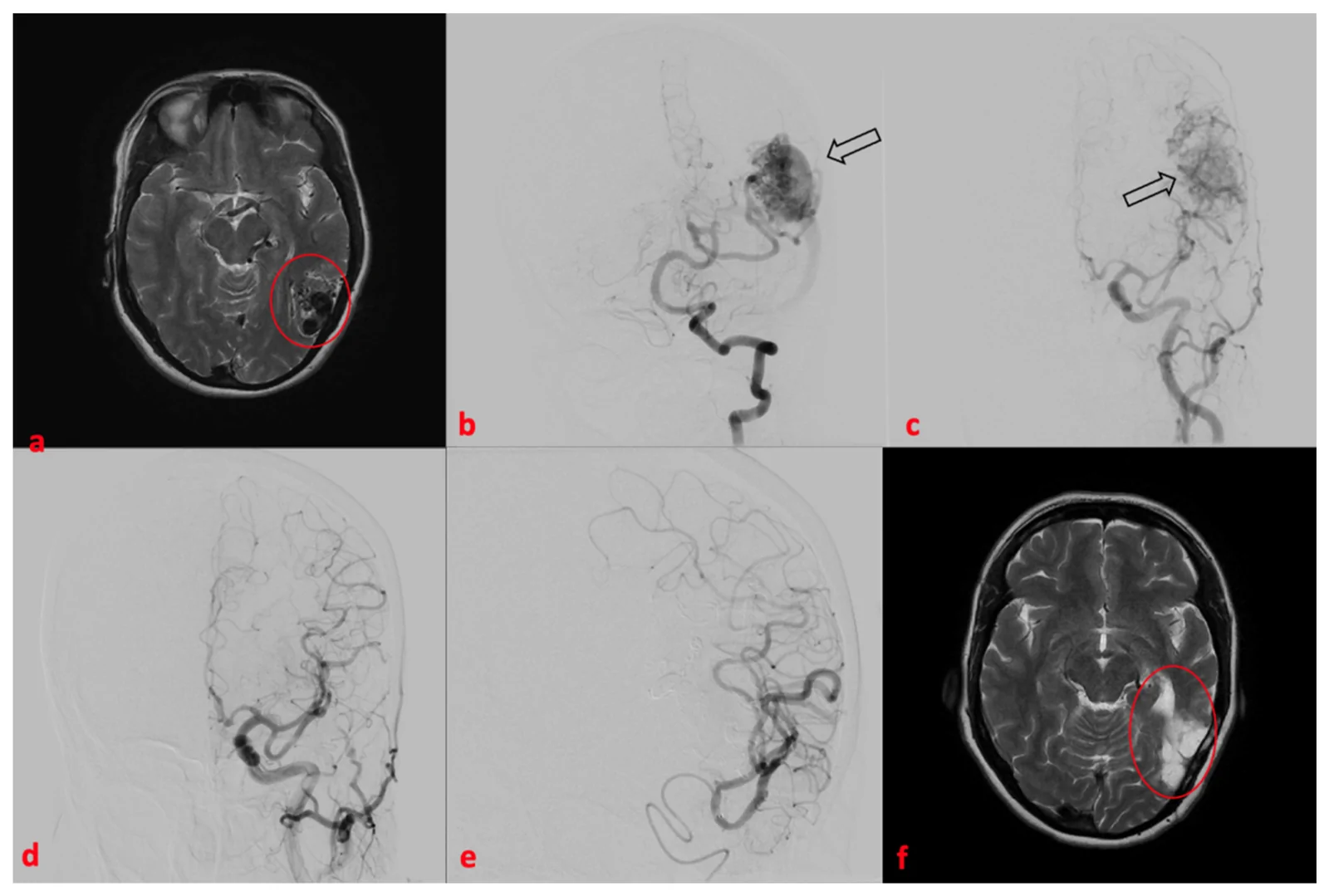
Endovascular-only case demonstrating pre- and post-treatment angiographic findings. Staged endovascular treatment of a left temporo-occipital arteriovenous malformation in a patient managed without same-session surgery. (**a**) Pre-treatment axial T2-weighted MRI at the level of the lateral ventricles. Serpiginous flow voids of the nidus are seen in the left temporo-occipital region (red circle), without mass effect, midline shift or hemorrhage. (**b**) Pre-treatment DSA, left vertebral artery injection, lateral projection, arterial phase. The compact nidus (open arrow) is supplied by posterior cerebral artery branches, with early venous filling in the arterial phase. (**c**) Pre-treatment DSA, left internal carotid artery injection, anteroposterior projection, arterial phase. Additional supply to the same nidus from the anterior circulation (open arrow) establishes dual arterial supply. (**d**,**e**) DSA after completion of staged embolization, left internal carotid (**d**) and left vertebral (**e**) injections. The anterior circulation supply shown in (**c**) and the nidus opacified in (**b**) no longer fill, and no residual shunt or early draining vein is identified from either territory. Normal posterior circulation branches remain patent. (**f**) Follow-up axial T2-weighted MRI one year after treatment, at a level comparable to (**a**). The previous flow voids (red circle) are replaced by high T2 signal with volume loss at the site of the treated malformation.

**Figure 3:**
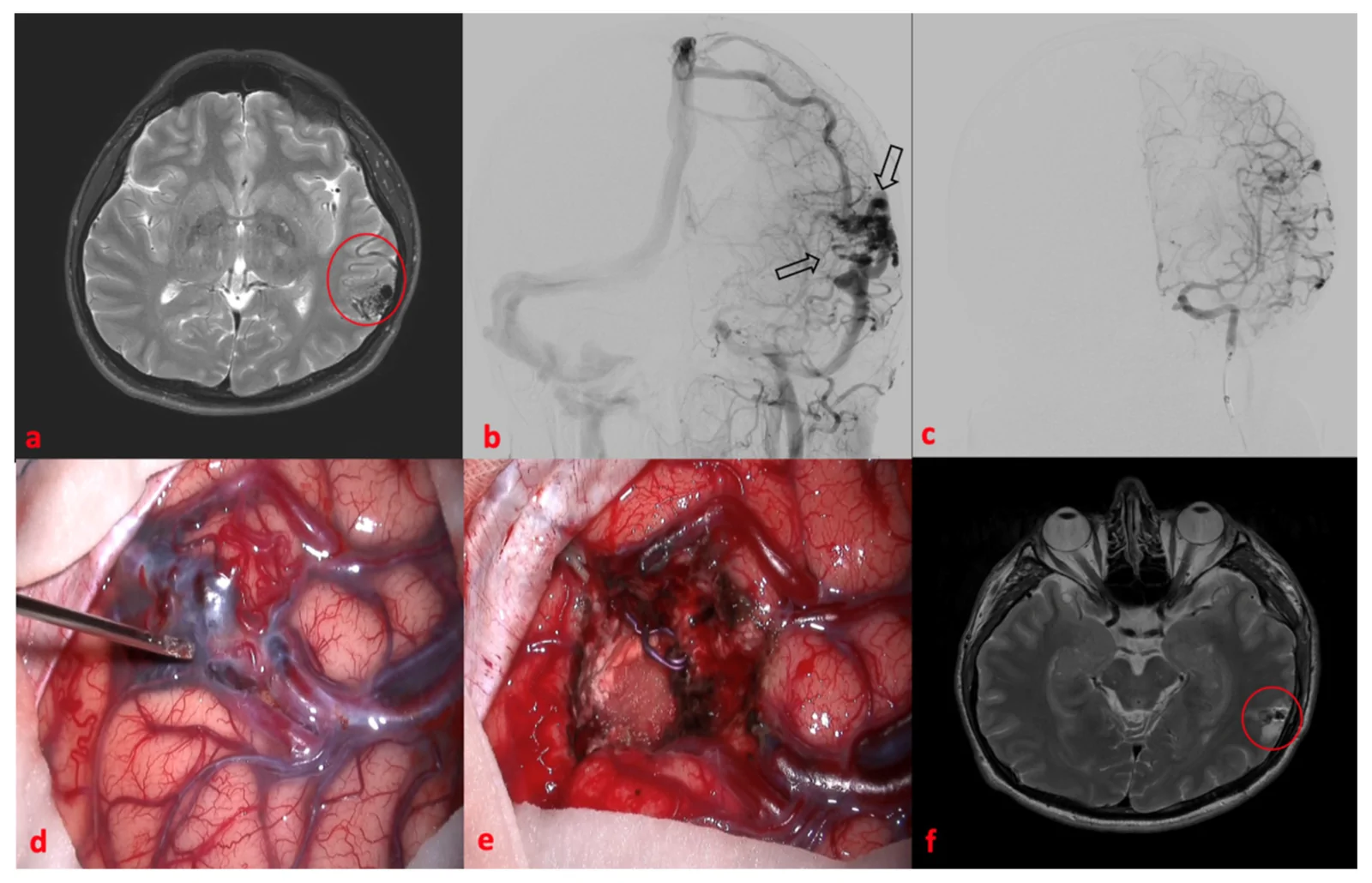
Hybrid case demonstrating pre-treatment angiography, post-embolization appearance, and complete nidus obliteration after microsurgical resection. Same-session hybrid embolization and microsurgical resection of a left temporal arteriovenous malformation. (**a**) Pre-treatment axial T2-weighted MRI. Serpiginous flow voids of the nidus are seen in the left temporal region (red circle). The lesion is cortical and superficial. (**b**) Pre-treatment DSA, left internal carotid artery injection, lateral projection, arterial phase. Open arrows indicate the nidus and its principal middle cerebral artery feeding pedicles; early venous drainage is present in the arterial phase. (**c**) DSA after embolization and before craniotomy, with the same projection as (**b**), showing reduction in the volume of opacified nidal vessels and in arteriovenous shunting. (**d**) Intraoperative microscopic view after craniotomy and dural opening, before resection. Dilated arterialized draining veins and abnormal vascular channels are visible on the cortical surface; the tip of a microsurgical instrument is in view at upper left. (**e**) Intraoperative microscopic view after complete resection, showing the resection cavity with hemostasis achieved and surrounding cortical vessels preserved. Dark material at the cavity margin is embolic agent within the excised nidal bed. (**f**) Follow-up axial T2-weighted MRI one year after treatment. Postoperative change with a small gliotic area is seen at the resection site (red circle). No flow voids and no residual or recurrent malformation are present.

The 10 endovascular-only patients with residual nidus went on to radiosurgery or surveillance, and their eventual status is not captured here. Figure 2 and Figure 3 show representative cases.

### 3.4. Complications and Mortality

Table 2 gives adverse events, each counted once, with mortality rows identifying which were fatal.

Treatment-related complications occurred in three of 19 hybrid patients (15.8%) and three of 24 endovascular-only patients (12.5%), with a risk difference of 3.3 percentage points (95% CI −17.9 to +26.5) and *p* = 1.000. Hemorrhage accounted for all three hybrid events and two of three endovascular-only events (*p* = 0.640); the third was a catheter-related dissection that resolved with a favorable outcome.

All four deaths in the series were in the hybrid group (4/19, 21.1%, against 0/24; risk difference of 21.1 points, 95% CI 2.4–43.3; *p* = 0.031), with a Kaplan–Meier survival difference (log-rank *p* = 0.029). Three of the four were the peri-procedural hemorrhages already counted among complications, not additional events. The fourth followed an uncomplicated resection.

Of the two hemorrhagic events in the endovascular-only group, one occurred between staged sessions and one after a single completed session, giving one of 24 (4.2%). With only one event, no precise estimate can be derived.

Clinical characteristics of the four fatal cases are summarized below. All four lesions were supratentorial with deep venous drainage; within the hybrid group, four of 13 patients with deep drainage died against 0 of six without, a comparison whose interval is uninformative (Firth odds ratio of 5.7, 95% CI 0.6–774). Three of the four were grade III, and three of the four were treated before 2019.

### 3.5. Functional Outcome

A favorable outcome (mRS 0–2) was reached in 13 of 19 hybrid patients (68.4%) and 18 of 24 endovascular-only patients (75.0%), with a risk difference of −6.6 percentage points (95% CI −32.5 to +19.1) and *p* = 0.738, which were unchanged after adjustment for age and grade. That interval runs from about 33 percentage points favoring endovascular treatment to about 19 favoring hybrid, and it is not evidence of equivalence.

### 3.6. Adjusted Analyses

Table 3 summarizes the exploratory Firth-adjusted analyses. Complete obliteration remained associated with hybrid treatment after adjustment for calendar period (odds ratio 41.88, 95% CI 3.43–6515; *p* = 0.001) and after adjustment for age and Spetzler–Martin grade (odds ratio 91.44, 95% CI 6.21–17567; *p* < 0.001), against an unadjusted odds ratio of 28.24. Calendar period alone was not associated with obliteration (odds ratio 1.98, 95% CI 0.45–11.74; *p* = 0.380), which is unsurprising since the angiographic advantage follows largely from the hybrid strategy including surgical excision.

For mortality, adjustment moved the estimate away from the null and rather than toward it: an unadjusted odds ratio of 14.23 (95% CI 1.37–1935) became 27.52 (95% CI 2.24–3913; *p* = 0.007) after adjustment for calendar period. These figures should not be taken at face value, since every model runs at an events-per-variable ratio of 4 or below and the intervals span three orders of magnitude. Functional outcome was unchanged by adjustment for age and grade (odds ratio 0.58, 95% CI 0.14–2.34; *p* = 0.447). We report the adjusted models because the unadjusted associations survive adjustment, not as causal estimates.

Propensity-score analyses were performed as sensitivity analyses. Because the positivity assumption was violated, these results are presented only in the Table 1, Table 2, Table 3 and Table 4 and were not used to support the main conclusions.

Within the hybrid group, outcomes differed between eras. Mortality was three of eight (37.5%) before 2019 and one of 11 (9.1%) afterwards (*p* = 0.262), while favorable functional outcome rose from 37.5% to 90.9% (*p* = 0.041), and obliteration was complete in every patient in both periods (Table 4). The mortality trend points the expected way but is not significant on four events, and both analyses are post hoc.

## 4. Discussion

Most published accounts of same-session hybrid treatment report the hybrid cohort alone or against historical microsurgical controls, and nearly all conclude favorably [10,11,12]. Three things here are less commonly available. The hybrid cohort is reported alongside the endovascular pathway it displaced within one institution, under the same records and outcome definitions. The confounding such a design carries is measured rather than mentioned. And the study reports an unfavorable safety signal, 21.1% mortality, well outside the published range, with each fatal event examined instead of buried in a summary statistic. Series with poor results are published less readily than series with good ones, so if the literature holds only favorable hybrid cohorts it will overstate how safe the technique is.

Obliteration in every hybrid patient is consistent with microsurgical series [3] and with recent hybrid and combined-modality series reporting 96% to 100% [13,14]. In comparison, the 58.3% after endovascular-only treatment sits inside the 45% to 61% range for embolization with curative intent [6].

That gap should not be over-read, because the endpoint is not symmetric. The hybrid strategy is defined by surgical removal of the nidus, so obliteration describes the procedure rather than an uncertain outcome. At the same time, the endovascular-only group contains patients for whom embolization was never meant to be curative alone and for whom the six-month angiogram was a planned interim look. The comparison therefore sets a completed strategy against one that, for a substantial minority, was still running. What it does show is what each pathway had achieved at six months, which matters clinically because residual nidus leaves a patient exposed during the radiosurgical latency period [4]. It says little about relative curative capacity under full multimodality treatment, and no statistical adjustment can repair an asymmetry of this kind.

Four deaths in 19 hybrid patients gives 21.1%, against 1% to 5% for elective AVM surgery in low- and moderate-grade lesions [3] and low single figures in published hybrid series. This is the finding that needs explaining most. We cannot explain it confidently.

Grade III-IV lesions were similarly distributed across the hybrid and endovascular-only groups (52.6% vs. 50.0%), and no major angioarchitectural characteristic differed significantly between treatment strategies. Therefore, differences in baseline AVM morphology alone are unlikely to account for the observed 21.1% mortality in the hybrid group.

Three observations deserve consideration. The first is timing: three of the four deaths fell between 2013 and 2016, before the standardized same-session protocol was introduced, and mortality among hybrid patients was 37.5% before 2019 against 9.1% after, with favorable functional outcome rising from 37.5% to 90.9%. A learning-curve effect is the reading we favor, with caveats: the mortality comparison is not significant, the analysis is post hoc, and the same boundary separates two eras of anesthetic and critical care practice as well as two levels of surgical experience.

The second is deep venous drainage, present in all four who died. It predicts spontaneous hemorrhage [2] and plausibly also marks surgical difficulty, since deep feeders are hardest to control early, but we raise it only as a question for larger series.

The third is sample size. Given the small number of procedures, mortality estimates remain unstable and should be interpreted cautiously: the 95% confidence interval around 21.1% runs from 8.5% to 43.3%. Our result is compatible with both a genuinely raised risk during early experience and chance. A multicenter propensity-matched study found preoperative embolization did not disproportionately increase surgical mortality after adjustment, at 4.9% against 2.1% [15].

Complication rates were similar between groups, at 15.8% against 12.5%, which are within the range reported for large embolization series [16].

Inter-session hemorrhage occurred in one of 24 endovascular-only patients, and a single event supports no inference. The concern is nonetheless real and documented elsewhere, since a partly embolized nidus is exposed to redistributed arterial flow and peri-procedural hemorrhage has been quantified in meta-analysis [9]. Arguing that a single-session strategy closes this window remains reasonable, but the argument rests on mechanism and published data rather than on ours. Closing the interval also relocates risk more than it removes it: all three hemorrhagic events in our hybrid group happened intraoperatively or immediately after.

Normal perfusion pressure breakthrough, first described by Spetzler and colleagues [17] and recently revisited in the endovascular setting by Theiss and Alaraj [18], offers a common frame: chronically dilated feeding arteries lose autoregulatory capacity, so abruptly removing the shunt exposes surrounding brain to a sudden rise in perfusion pressure. Embolization before resection may soften that shift without abolishing it, while staged embolization allows gradual adaptation at the price of the altered flow state that predisposes to bleeding between sessions.

A retrospective comparison of 19 hybrid patients with 24 endovascular-only patients, allocated non-randomly across two eras, cannot establish who should receive which treatment, so what follows is hypothesis rather than recommendation. The pattern fits a view in which same-session hybrid treatment delivers reliable immediate angiographic cure at the cost of concentrating risk into one procedure, a cost that appeared to fall as institutional experience accumulated. If that reading is right, the question for a center considering the technique is not whether hybrid treatment is better in general but whether its own volume and team experience suffice to get through the early part of the curve safely. Guidance from the AHA/ASA, the Society of NeuroInterventional Surgery and the European consensus conference converges on individualized multidisciplinary decision-making and names no single optimal modality [7,19,20].

The design is retrospective, single-center and non-randomized, with allocation by clinical judgment and institutional capability rather than protocol. Patients offered hybrid treatment were those judged to have a resectable nidus and to be fit for craniotomy, drawing on information our dataset does not hold in structured form. Every hybrid lesion was supratentorial while all five infratentorial lesions went to embolization, hybrid patients were eleven years younger at the median and comorbidity was never coded uniformly, so residual confounding by baseline health remains and favors the hybrid group.

Treatment strategy is also confounded with calendar period beyond what this cohort can resolve. Comparing the groups partly compares practice in 2014 with practice in 2021: different embolic agents and microcatheters, different neuroanesthesia and intensive care, different imaging, and different thresholds for intervening at all, particularly after ARUBA shifted attitudes to unruptured lesions [8]. Models holding both treatment and period gave stable estimates, but with this collinearity they show only that the association persists when period is included.

The sample is small and the events fewer still. Four deaths drive the whole mortality analysis, every multivariable model sits below the conventional events-per-variable minimum, and both principal comparisons show complete separation, so odds ratio intervals span orders of magnitude; the null functional outcome result is therefore not evidence of equivalence. Follow-up duration was missing for five patients, and a median of 41 to 53 months may be too short to catch late recurrence or delayed rebleeding. Outcomes came from clinical records without independent adjudication or blinding, and multiple comparisons went uncorrected, so the two findings nearest the threshold are provisional. Results from one center during a period of technical transition may not describe a center with different volume or a mature program.

A randomized comparison faces the difficulties ARUBA illustrated [8]. Multicenter propensity-matched registries with prespecified outcome definitions, prospectively collected comorbidities and explicit recording of each institution’s cumulative hybrid experience offer a more achievable route and should report the calendar period of every procedure, which our data suggest may be as informative as any clinical covariate.

## 5. Conclusions

In this small single-center cohort, same-session hybrid treatment achieved complete angiographic obliteration in every case but accounted for all four deaths, while functional outcomes did not differ from endovascular-only treatment. Because the groups were selected for different clinical purposes and treated predominantly in different periods, these associations are hypothesis-generating rather than a basis for changing practice, and they mark operator experience and calendar period as variables that future comparative studies of hybrid AVM treatment will need to measure and report.

## Figures and Tables

**Table 1:** Baseline characteristics, AVM angioarchitecture, treatment parameters and calendar period.

Variable	Hybrid (*n* = 19)	Endovascular (*n* = 24)	*p*
Demographic and clinical characteristics			
Age, years, median [IQR]	31.0 [18.5–37.0]	42.5 [32.8–52.0]	0.040
Male sex, *n* (%)	13 (68.4)	18 (75.0)	0.738
Baseline mRS > 2, *n* (%)	3 (15.8)	4 (16.7)	1.000
Presenting symptom, *n* (%)			
Headache	9 (47.4)	10 (41.7)	0.764
Seizure	2 (10.5)	1 (4.2)	0.575
Hemorrhage	7 (36.8)	10 (41.7)	1.000
Focal neurological deficit	3 (15.8)	3 (12.5)	1.000
AVM angioarchitectural features			
SM grade, median [IQR]	3.0 [2.0–3.0]	2.5 [2.0–3.0]	0.750
Grade I, *n* (%)	1 (5.3)	1 (4.2)	-
Grade II, *n* (%)	8 (42.1)	11 (45.8)	-
Grade III, *n* (%)	7 (36.8)	10 (41.7)	-
Grade IV, *n* (%)	3 (15.8)	2 (8.3)	-
Grade III–IV (high grade), *n* (%)	10 (52.6)	12 (50.0)	1.000
Supratentorial location, *n* (%)	19 (100.0)	19 (79.2)	0.056
Nidus diameter ≥ 3 cm, *n* (%)	13 (68.4)	12 (50.0)	0.351
Eloquent location, *n* (%)	11 (57.9)	16 (66.7)	0.752
Deep venous drainage, *n* (%)	13 (68.4)	15 (62.5)	0.755
Associated aneurysm, *n* (%)	8 (42.1)	6 (25.0)	0.329
Treatment parameters			
EVOH alone, *n* (%)	16 (84.2)	18 (75.0)	-
nBCA alone, *n* (%)	0 (0.0)	2 (8.3)	-
EVOH + nBCA, *n* (%)	3 (15.8)	4 (16.7)	-
Embolization sessions, median [IQR]	1.0 [1.0–1.0]	1.5 [1.0–3.0]	0.011
More than one session, *n* (%)	3 (15.8)	12 (50.0)	0.026
Hospital stay, days, median [IQR]	10.0 [6.0–14.5]	3.0 [2.0–6.5]	0.004
Follow-up, months, median [IQR]	41.5 [14.5–65.2]	53.0 [16.5–83.2]	0.413
Calendar period			
Treated 2012–2018, *n* (%)	8 (42.1)	21 (87.5)	0.003
Treated 2019–2024, *n* (%)	11 (57.9)	3 (12.5)	0.003
Year of treatment, median	2019	2014	-

Abbreviations: AVM, arteriovenous malformation; EVOH, ethylene-vinyl alcohol copolymer; IQR, interquartile range; mRS, modified Rankin Scale; nBCA, *n-butyl cyanoacrylate*; SM, Spetzler–Martin. Statistical methods: Continuous variables were compared using the Mann–Whitney U test and categorical variables using Fisher’s exact test.

**Table 2:** Angiographic, safety and functional outcomes.

Outcome	Hybrid (*n* = 19), *n* (%) [95% CI]	Endovascular (*n* = 24), *n* (%) [95% CI]	Risk Difference, pp [95% CI]	*p*
Complete angiographic obliteration at 6 months	19 (100.0) [83.2–100.0]	14 (58.3) [38.8–75.5]	+41.7 [+17.6 to +61.2]	0.001
All-cause mortality	4 (21.1) [8.5–43.3]	0 (0.0) [0.0–13.8]	+21.1 [+2.4 to +43.3]	0.031
Any treatment-related complication	3 (15.8) [5.5–37.6]	3 (12.5) [4.3–31.0]	+3.3 [−17.9 to +26.5]	1.000
Hemorrhagic complication	3 (15.8) [5.5–37.6]	2 (8.3) [2.3–25.8]	+7.5 [−12.8 to +30.0]	0.640
Catheter-related arterial dissection	0 (0.0) [0.0–16.8]	1 (4.2) [0.7–20.2]	−4.2 [−20.2 to +13.0]	1.000
Hemorrhage between staged sessions	0 (0.0) [0.0–16.8]	1 (4.2) [0.7–20.2]	−4.2 [−20.2 to +13.0]	1.000
Fatal events	4 (21.1) [8.5–43.3]	0 (0.0) [0.0–13.8]	+21.1 [+2.4 to +43.3]	0.031
Peri-procedural hemorrhage	3 (15.8) [5.5–37.6]	0 (0.0) [0.0–13.8]	+15.8 [−1.4 to +37.6]	0.079
Late medical complication	1 (5.3) [0.9–24.6]	0 (0.0) [0.0–13.8]	+5.3 [−9.2 to +24.6]	0.442
Favorable functional outcome (mRS 0–2)	13 (68.4) [46.0–84.6]	18 (75.0) [55.1–88.0]	−6.6 [−32.5 to +19.1]	0.738
Unfavorable outcome or death (mRS > 2)	6 (31.6) [15.4–54.0]	6 (25.0) [12.0–44.9]	+6.6 [−19.1 to +32.5]	0.738
Overall survival (Kaplan–Meier)	4 events	0 events	-	0.029

Abbreviations: CI, confidence interval; mRS, modified Rankin Scale; pp, percentage points. Statistical methods: Confidence intervals for proportions and risk differences were calculated using Wilson and Newcombe methods, respectively. Group comparisons were performed using Fisher’s exact test or the log-rank test, as appropriate.

**Table 3:** Exploratory adjusted analyses.

Outcome and Model	Odds Ratio [95% CI]	*p*
Complete angiographic obliteration		
Unadjusted	28.24 [3.17–3742]	0.001
Adjusted for calendar period	41.88 [3.43–6515]	0.001
Adjusted for age and SM grade	91.44 [6.21–17,567]	<0.001
Calendar period alone (2019–2024 vs. 2012–2018)	1.98 [0.45–11.74]	0.380
All-cause mortality		
Unadjusted	14.23 [1.37–1935]	0.031
Adjusted for calendar period	27.52 [2.24–3913]	0.007
Favorable functional outcome (mRS 0–2)		
Unadjusted	0.72 [0.19–2.75]	0.738
Adjusted for age and SM grade	0.58 [0.14–2.34]	0.447
Within the hybrid group: effect of calendar period		
Mortality, 2019–2024 vs. 2012–2018 (1/11 vs. 3/8)	0.22 [0.02–1.78]	0.159
Favorable outcome, 2019–2024 vs. 2012–2018 (10/11 vs. 3/8)	-	0.041
Complete obliteration, 2019–2024 vs. 2012–2018 (11/11 vs. 8/8)	-	1.000

Abbreviations: CI, confidence interval; mRS, modified Rankin Scale; SM, Spetzler–Martin. Statistical methods: Odds ratios were estimated using Firth-penalized logistic regression because of sparse events and complete separation. These adjusted analyses are exploratory and should be interpreted cautiously given the small sample size.

**Table 4:** Individual description of the four fatal events, all in the hybrid group.

	Case 1	Case 2	Case 3	Case 4
Year of treatment	2014	2019	2016	2014
Treated before/after standardized protocol	Before (2012–2018)	After (2019–2024)	Before (2012–2018)	Before (2012–2018)
Age, years	36	49	38	60
Sex	Female	Male	Male	Male
SM grade	III	III	III	II
Location	Supratentorial	Supratentorial	Supratentorial	Supratentorial
Nidus diameter ≥ 3 cm	Yes	No	Yes	Yes
Eloquent location	Yes	Yes	No	Yes
Deep venous drainage	Yes	Yes	Yes	Yes
Associated aneurysm	Yes	Yes	No	No
Presenting symptom	Headache	Focal deficit	Headache	Headache
Baseline mRS > 2	No	Yes	No	No
Complete obliteration achieved	Yes	Yes	Yes	Yes
Treatment-related complication	Peri-procedural hemorrhage	Peri-procedural hemorrhage	Peri-procedural hemorrhage	Hospital-acquired infection
Length of stay, days	Same day	37	3	12
Timing of death	Day of procedure	During index admission	Day 3	Day 12
Cause of death	Peri-procedural hemorrhage	Peri-procedural hemorrhage	Peri-procedural hemorrhage	Sepsis secondary to hospital-acquired pneumonia

Abbreviations: mRS, modified Rankin Scale; SM, Spetzler–Martin.

## Data Availability

The data presented in this study are available from the corresponding author upon reasonable request. The data are not publicly available due to patient privacy and ethical restrictions.
